# Seroepidemiological feature of Q fever among sheep in Northern Iran

**DOI:** 10.1186/1742-4690-9-S1-P40

**Published:** 2012-05-25

**Authors:** Ehasn Mostafavi, Saber Esmaeili, Mahin Shahdordizadeh, Hadi Mahmoudi, Hamid Liriayii, Fahimeh Bagheri Amiri

**Affiliations:** 1 Department of Epidemiology at Pasteur Institute of Iran, Tehran, Iran

## Introduction and aims

Q fever is a zoonosis caused by Coxiella burnetii, which infects various hosts, including humans and animals. As Q fever is considered an important factor in public health, and there is little epidemiological information on the status of the disease in various parts of Iran, this study has been carried out to evaluate the seroepidemiology of Q fever among sheep in the province of Mazandaran, northern Iran.

## Materials and methods

In this study, samples from sheep were collected from western, central and eastern regions of Mazandaran in 2010-2011. Serum samples were analyzed by ELISA test.

## Results

In this study, 253 serum samples were collected. The infection rate with Q fever was 23.7%. The chi-square test showed a significant statistical relationship between central (33.8%) and eastern (27.2%) regions compared to western regions (8.5%). There was no significant difference between the three groups of sheep with respect to age. No significant statistical relationship was seen between infection rate and age and gender.

## Discussion

The infection rate of coxiella burnetii in this study, is similar to the results of other research carried out in various parts of the country. With respect to the fact that there is a higher infection rate in the eastern and central regions of the province, compared to the western region, and also in imported animals from Afghanistan, the hypothesis that the disease is spreading from eastern boundaries becomes more probable. It is recommended that complementary research be carried out on other animals, on high-risk persons and on ticks, in order to reveal the status of the disease in the province.

